# Murine hepatoma treatment with mature dendritic cells stimulated by *Trichinella spiralis* excretory/secretory products

**DOI:** 10.1051/parasite/2020045

**Published:** 2020-07-21

**Authors:** Jing Ding, Xiaolei Liu, Bin Tang, Xue Bai, Yang Wang, Shicun Li, Jian Li, Mingyuan Liu, Xuelin Wang

**Affiliations:** 1 Key Laboratory for Zoonoses Research, Ministry of Education, Institute of Zoonoses, College of Veterinary Medicine, Jilin University, OIE Collaborating Center on Foodborne Parasites in Asian-Pacific Region Changchun 130062 P.R. China

**Keywords:** *Trichinella spiralis*, excretory/secretory products, dendritic cells, H22

## Abstract

Excretory/Secretory Products (ESPs) of the nematode *Trichinella spiralis* contain antitumor-active substances that inhibit tumor growth. Mature dendritic cells (DCs) play a critical role in the antitumor immunity of the organism. As pathogen-derived products, it ought to be discussed whether *T. spiralis* ESPs will reduce the antitumor effect of mature DCs from the host before it is applied to patients’ tumors. Therefore, the aim of this work was to evaluate the immunological effect of DCs stimulated by *T. spiralis* ESPs in H22 tumor-bearing mice. H22 tumor model mice in this study were randomly divided into four groups according to the treatment: PBS control group, ESP group, DCs group, and DCs stimulated with *T. spiralis* ESP (ESP+DCs group). The antitumor effect was evaluated by tumor inhibition rate and cytokine detection using ELISA. The results showed significant inhibition in tumor growth in the ESP+DCs, DCs and ESP groups when compared with the PBS control group (*p* < 0.01, *p* < 0.01, and *p* < 0.05, respectively). However, no significant difference was observed on tumor inhibition rates between the ESP+DCs and DCs groups. The decrease in IL-4, IL-6, and IL-10, and the increase in IFN-γ between the DCs and ESP+DCs groups were also not significant. Therefore, DCs stimulated by ESP did not reduce the antitumor effect of mature DCs, which demonstrated that the *T. spiralis* ESP would not affect the antitumor effect of mature DCs by modulating the immune response of the host, and that ESPs are safe in antitumor immunology when applied in a tumor model mice.

## Abstract (fr)

Les produits d’excrétion-sécrétion (PES) du nématode *Trichinella spiralis* contiennent des substances actives antitumorales qui inhibent la croissance tumorale. Les cellules dendritiques (CD) mûres jouent un rôle essentiel dans l’immunité anti-tumorale de l’organisme. En tant que produits dérivés d’agents pathogènes, il convient de déterminer si les PES de *T. spiralis* réduisent l’effet antitumoral des CD mûrs de l’hôte, avant leur application aux tumeurs des patients. Par conséquent, l’objectif de ce travail était d’évaluer l’effet immunologique des CD stimulées par les PES de *T. spiralis* chez des souris porteuses de tumeurs H22. Les souris modèles à tumeurs H22 dans cette étude ont été réparties au hasard en quatre groupes selon le traitement : groupe témoin PBS, groupe PES, groupe CD et groupe CD stimulés par les PES de *T. spiralis* (CD+PES). L’effet antitumoral a été évalué par le taux d’inhibition des tumeurs et la détection des cytokines en utilisant un dosage ELISA. Les résultats ont montré une inhibition significative de la croissance tumorale dans les groupes CD+PES, CD et PES par rapport au groupe témoin PBS (*p* < 0,01, *p* < 0,01 et *p* < 0,05, respectivement). Cependant, aucune différence significative n’a été observée sur le taux d’inhibition de la tumeur entre les groupes CD+PES et CD. La diminution de l’IL-4, de l’IL-6, de l’IL-10 et l’augmentation de l’IFN-γ entre les groupes CD et CD+PES n’étaient pas non plus significatives. Ainsi, les CD stimulées par les PES n’ont pas réduit l’effet antitumoral des CD mûrs, ce qui a démontré que les PES de *T. spiralis* n’affectaient pas l’effet antitumoral des CD mûrs en modulant la réponse immunitaire de l’hôte et que les PES sont sûrs en immunologie antitumorale quand ils sont appliqués à des souris modèles tumorales.

## Introduction

*Trichinella spiralis* was recognized for the first time in 1977 as a nematode that can negatively influence tumor growth and prolong the lifespan of tumor-bearing mice [27]. Wang et al. demonstrated a strong antiproliferative and pro-apoptotic effect of *T. spiralis* antigens on two different cell lines (K562 and H7402) *in vitro* [45]. Even in the case of a very aggressive tumor such as melanoma, *T. spiralis* infection is effective not only in reducing tumor growth but also against malignant cell dissemination [16, 27].

A certain number of the studies available on the anti-tumor mechanism of *T. spiralis* focus on the immunomodulatory effects of its antigens. Kang revealed that CXCL9, CXCL10, IL-4, CXCL1, and CXCL13 expression may be related to tumor regression in mice with *T. spiralis* infection [16]. *Trichinella spiralis* antigens induce a significant decrease in serum IL-17, a significant increase in serum IL-10, and an increased percentage of splenic CD4+T-cells and intestinal FoxP3+ Treg cells as a defense against colon cancer in a murine model [9]. The immunomodulatory effect is based on the development and maintenance of Th2 response during *T. spiralis* infection and different from the anti-tumor immunomodulatory effects of mature dendritic cells (DCs) in the organism.

Mature DCs are correlated with an immune contexture characterized by TH1 polarization, infiltration by effectors cells (T cells, NK cells, and plasma cells) and cytotoxic effector functions [36, 40]. Mature DCs play a critical role in coordinating cellular interplay and in the anti-tumor immunity in host defense against pathogens and malignantly transformed cells [10, 17–19, 25, 30, 32]. Pathogen-derived products have the ability to induce the maturation of bone marrow-derived dendritic cells (BMDCs) [12]. ESPs of *T. spiralis* are a complex mixture of different molecules with different biological activities, which help in *T. spiralis* long-term survival by successfully evading and modulating host immunity [5]. The effect of *T. spiralis* on cells and molecules of the host’s immune system is achieved through ESPs [34]. *Trichinella spiralis* ESPs contain antitumor-active substances that inhibit tumor growth [24, 44, 45]. ESPs of *T. spiralis* muscle larvae (ML) can induce the transformation of rat bone marrow-derived dendritic cells (BMDCs) to semi-matured status DCs [5]. Semi-matured DCs can be potentiated by either tolerogenic or pro-tumorigenic responses [8]. As pathogen-derived products, it ought to be discussed whether *T. spiralis* ESPs will reduce the antitumor effect of mature DCs from the host before it is applied to patients’ tumors. However, a study on the immune safety of mature DCs stimulated by ESPs of *T. spiralis* against tumors is still lacking. Therefore, it is of utmost importance to evaluate the antitumor effect of *T. spiralis* ESPs.

## Materials and methods

### Ethical standards

Experimental animals used in this study were purchased from the Jilin University experimental animal center, China. To establish *T. spiralis* infection, ML were collected from infected experimental mice to ensure the maintenance of the life cycle system. *Trichinella spiralis* species were preserved in the Food-Borne Parasitology Laboratory of Key Laboratory for Zoonoses Research, Ministry of Education, Institute of Zoonoses, Jilin University, and genotyped by the OIE Collaborating Center on Foodborne Parasites in the Asian-Pacific Region. All experimental procedures were reviewed and approved by the Ethical Committee of Jilin University for the Care and Use of Laboratory Animals.

### Animals, parasites, cells, and antibodies

*Trichinella spiralis* (ISS534) were obtained from specific pathogen-free female inbred Wistar rats (220.0 ± 20.0 g), maintained by serial oral passage. Eight-week-old BALB/c and C57BL/6 male mice (22.0 ± 2.0 g) were housed under proper care and specific pathogen-free conditions by the institutional guidelines. Wistar rats, BALB/c and C57BL/6 mice were purchased from Jilin University Experimental Animal Center (Jilin, China), with certificate No. 2016-0001 in conformity with SCXK (Jilin). The H22 hepatoma cell line was purchased from Bio-Rad Life Sciences Development Co., Ltd. (Beijing, China). FITC anti-mouse CD11c, PE Anti-Mouse MHC Class II (I-A), and PE anti-mouse CD86 were purchased from eBioscience (USA).

### Preparation of *Trichinella spiralis* antigen

*Trichinella spiralis* ESPs were collected as previously described [20]. Briefly, *T. spiralis* ML were isolated from the muscle tissue of Wistar rats by the artificial digestion method. Wistar rats were infected with the isolated ML by intragastric administration to harvest adults (ADs) and newborn larvae (NBL) of *T. spiralis*. At 6 days post-infection (dpi), ADs of *T. spiralis* were harvested from the intestine of the infected Wistar rats, and the NBL were harvested from the culture medium after the AD were cultured in RPMI 1640 culture medium (Gibco, USA) overnight. The isolated ADs and NBL of *T. spiralis* were cultured in a Petri dish containing RPMI-1640 culture medium without fetal bovine serum (FBS) and incubated at 37 °C in a CO_2_ incubator for 24 h. The culture supernatant was collected by centrifugation, dialyzed, and concentrated. The protein concentration was measured by QuantiPro BCA Assay Kit (Sigma-Aldrich, USA) and the supernatant was stored at −80 °C until use. The mixture of ML, AD, and NBL supernatant was concentrated and used as *T. spiralis* ESP in this experiment.

### Isolation and culture of mice bone-marrow-derived DCs

Mouse BMDCs were obtained with an improved method that was previously described [22]. Briefly, bone marrow was collected from C57BL/6 mice, and erythrocytes were removed. BMDCs were cultured in RPMI 1640 medium supplemented with 10% fetal calf serum (FCS), 2 mM L-glutamine, 1 mM sodium pyruvate, 10 mM HEPES, 50 μM 2-mercaptoethanol (Sigma Aldrich, USA), and 50 U/mL gentamycin (Invitrogen, USA). On day 2, two-thirds of the original medium was replaced with fresh medium. On day 5, the floating cells were gently removed and the adherent cells were cultured with fresh medium containing 20 ng/mL GM-CSF, 20 ng/mL IL-4, and 10 ng/mL TNF-α. On day 8, the mature DCs were used for cell imaging experiments. Cells treated with phosphate-buffered saline (PBS) were used as a negative control [22, 39].

### Scanning Electron Microscopy (SEM) imaging

Lab-Tek 4-well Chambered Cover glasses (Nunc, Thermo Fisher Scientific) were used for cell imaging experiments. Glass coverslips were coated with Poly-Lysine (Sigma-Aldrich, USA) 400 μg/mL in pyrogen-free water and incubated for 1 h at 37 °C. A total amount of 3 × 10^5^ mature DCs were seeded in a Lab-Tek 4-well Chamber with coated coverslips on the bottom, and incubated at 37 °C for 5 h. After cell attachment to the coverslips, the cells were washed three times in PBS, fixed in 2.5% glutaraldehyde (Sigma-Aldrich, USA) for 2 h, then fixed in 2% osmium tetroxide (Sigma-Aldrich, USA) for 1 h. The specimens were further rinsed in PBS, then dehydrated in increasing concentrations of ethanol (10, 20, …, 90, 95, and 100%) for 10 min at each concentration, and finally air-dried overnight before analysis [3].

### FACS analysis

Cells were incubated with FITC-CD11c, PE-CD86, and PE-MHC-II for 30 min at 4 °C. All washing steps were performed in PBS or PBS supplemented with 2% FBS to avoid unspecific binding. Flow cytometry analyses were performed on a Coulter Epics XL flow cytometer (Beckman Coulter, Marseille, France) equipped with the analysis software Expo32 ADC (Beckman Coulter) [7].

### H22 cell culture

The H22 mouse hepatoma cell line was purchased from the American Type Culture Collection (Rockville, MD, USA) and stored at −80 °C after arrival. The H22 cells were cultured in DMEM supplemented with 10% FBS (v/v), 100 U/mL penicillin G (Sigma-Aldrich, USA), and 100 μg/mL streptomycin (Sigma-Aldrich, USA). The cultures were maintained in a humidified incubator at 37 °C and 5% CO_2_ [23].

### Model establishment, grouping, and administration methods

Forty-eight C57BL/6 male mice (each with a bodyweight of 20 ± 2 g) were selected. The mice were intraperitoneally treated with H22 cells (1 × 10^6^) under sterile conditions and were sacrificed after 5 days to collect ascites (the collected ascitic tumor fluid was thick and ivory in color, while the yellow or red ascitic fluid was discarded). Ascitic tumor fluid was then diluted with normal saline to adjust the cell concentration to 1 × 10^5^/mL. Each mouse was subcutaneously treated with 0.5 mL cell suspension in the right axilla of the forelimb. After inoculation, mice were randomly divided into four treatment groups: PBS control group, DCs, ESP and ESP+DCs therapy group (*N* = 12 per group). At 24 h after inoculation, the PBS control group was intraperitoneally treated with 2 mL/kg PBS once a day; the ESP therapy group was intraperitoneally treated with 50 μg/kg ESP once every 7 days; the DCs therapy group was intraperitoneally treated with 6 × 10^6^ mature DCs previously treated with PBS once every 7 days; the ESP+DCs therapy group was intraperitoneally treated with 6 × 10^6^ mature DCs previously treated with *T. spiralis* ESPs (containing 50 μg/mL protein) once every 7 days. The administration period was concluded after 21 days. Mice were euthanized the day after the end of the experiment and the subcutaneous tumor was collected [28].

### Body and tumor weight measurement

Mice body weight was measured every third day during the treatment period. At the same time points, the blood samples were collected from orbital sinus. The tumor was removed and weighed, and the tumor inhibition rate was calculated using the following formula [21, 31]:

$$\mathrm{Inhibition}\;\mathrm{rate}\;\left(\%\right)\;=\left(1-\frac{\mathrm{tumor}\;\mathrm{weight}\;\mathrm{of}\;\mathrm{therapy}\;\mathrm{group}}{\mathrm{tumor}\;\mathrm{weight}\;\mathrm{of}\;\mathrm{control}\;\mathrm{group}}\right)\times \;100\%$$

### ELISA

A blood sample from each mouse of all groups was collected as described [1]. Blood was centrifuged at 300× *g* for 10 min. The concentrations of the proinflammatory cytokines IL-4, IL-6, IL-10 and IFN-γ in the serum were assessed by ELISA (R&D Systems, USA), according to the manufacturer’s recommended protocols (eBioscience, USA).

### Statistical analysis

Statistical analysis was performed using SPSS 10.0 software (SPSS Inc., USA). Results were expressed as mean ± SD. Comparisons between the control and therapy groups were performed by one-way ANOVA. A value of *p* < 0.05 was considered statistically significant.

## Results

### SEM

**Figure 1:**
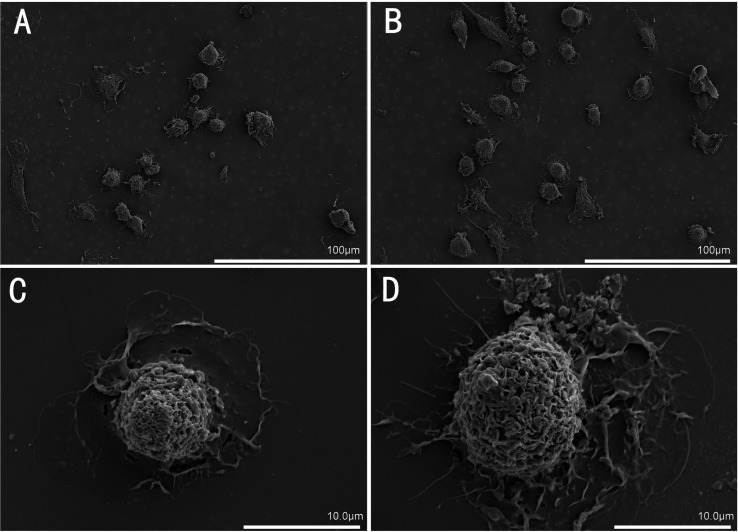
Bone marrow-derived dendritic cell (BMDC) morphology by scanning electron microscopy (SEM). (A) SEM image (500× magnification) of immature dendritic cells (DCs) formed by monocytes after 6 days of induction; (B) SEM image (500× magnification) of mature DCs formed by monocytes after 8 days of induction; (C) SEM image (4K× magnification) of immature DCs formed by monocytes after 6 days of induction; (D) SEM image (4K× magnification) of mature DCs formed by monocytes after 8 days of induction.

According to the morphology characterization of the typical mature DCs, the surface of DCs originating from bone marrow-derived monocytes after 8 days of induction appeared rough and intact under SEM, therefore, these cells were considered mature (Figs. 1B, 1D). Conversely, DCs induced from monocytes after 6 days of induction did not have the typical morphology of mature DCs and were considered immature (Figs. 1A, 1C).

### Flow cytometric analysis of DC

**Figure 2:**
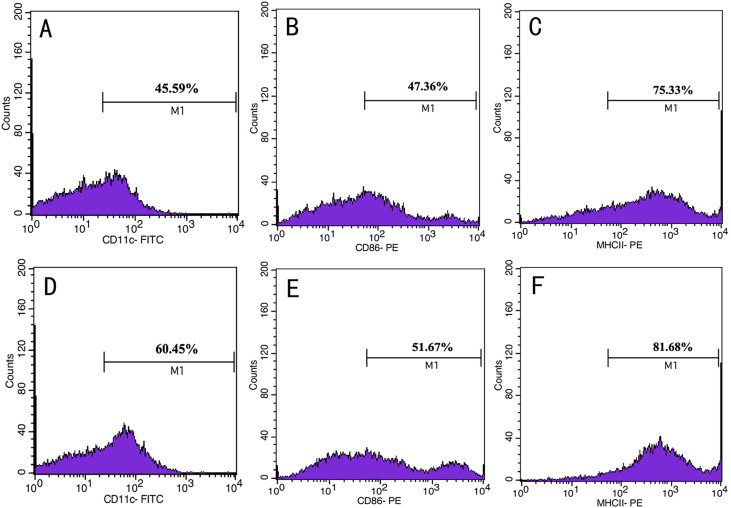
CD11c, CD86 and MHCII (I-A) expression in mature and immature dendritic cells (DCs). (A–C) CD11c, CD86 and MHCII (I-A) expression in immature DCs; (D–F) CD11c, CD86 and MHCII (I-A) expression in mature DCs.

The ratio of CD11c^+^ monocytes after 8 days of induction was 60.45%. The expression of the mature DC cell surface marker CD86 and MHCII family MHCII (I-A) was 51.67% and 81.68%, respectively (Figs. 2A–2C), while the ratio of CD11c^+^ cells and the expression of CD86 and MHCII (I-A) in monocytes after 6 days of induction was 45.59%, 47.36%, and 75.33%, respectively (Figs. 2D–2F), which was higher than that of cells after 8 days of induction. The expression of CD11c, a specific surface marker of BMDCs, indicated that mouse bone marrow-derived monocytes cultured *in vitro* could be induced into DCs in relatively high purity.

### The general condition of H22 tumor-bearing mice

**Figure 3:**
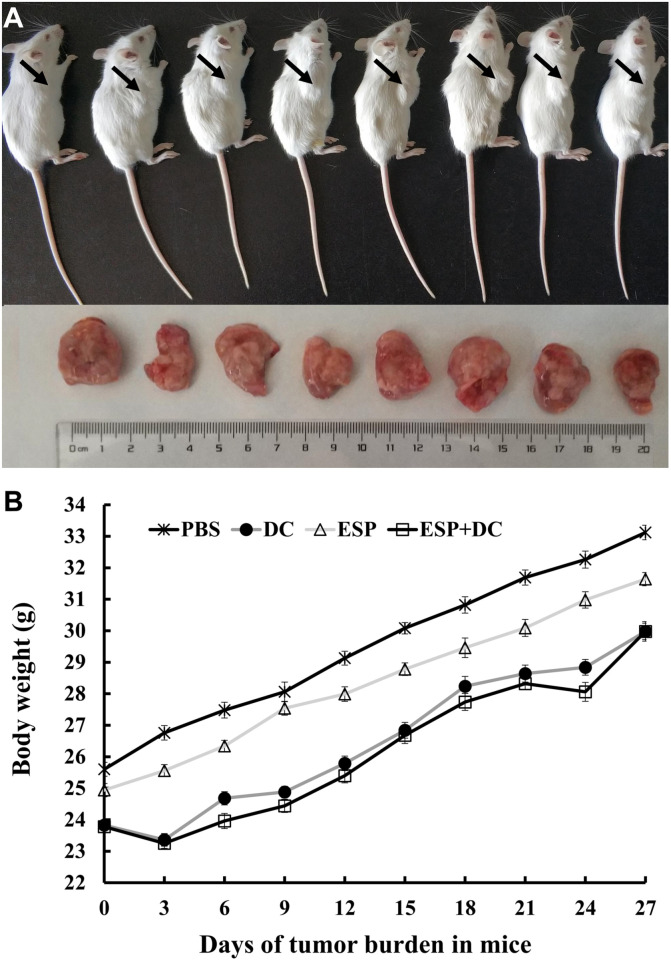
Body weight of each mice group. (A) H22 mouse hepatoma model; (B) Tumor growth curves of four H22-bearing mice groups with different stroma amounts and treated with PBS, excretory/secretory products (ESPs), immature dendritic cells (DCs) and ESP+DCs.

H22 cells can develop into tumors after subcutaneous injection in mice (Fig. 3A). Mean body weight at day 27 was significantly decreased in the ESP+DCs and DCs group compared with the ESP and PBS groups. However, this weight variation was not significantly different between the mice treated with DCs and DCs stimulated with ESPs. In the PBS group, the mean body weight was significantly increased compared to of all the other three groups (Fig. 3B).

### Tumor growth inhibition

Tumor inhibition rates in the ESP+DCs, DCs, and ESP groups were 48.82 ± 5.88%, 48.57 ± 6.06, and 34.78 ± 2.29%, respectively. Significant inhibition in tumor growth was observed in the ESP+DCs, DCs and ESP groups when compared with the PBS group (*p* < 0.01, *p* < 0.01 and *p* < 0.05, respectively). The inhibition rates in the ESP+DCs and DCs groups were significantly higher than those in the ESP group (*p* < 0.05) (Table 1). The inhibition rate in the ESP+DCs and DCs group was not significantly different.

**Table 1:** Tumor weights of BALB/c mice in each group and tumor inhibition rates.

Group	Average tumor weight/g	Inhibition rate (%)
PBS	3.70835 ± 0.58923	
ESP	2.41605 ± 0.58923	33.7405 ± 0
DCs	1.90088 ± 0.58923	47.6688 ± 0
ESP+DCs	1.8928 s ± 0.5892	47.948 s ± 0

Results are expressed as mean ± SD. *p < 0.05 (ESP *vs.* PBS control group); **p < 0.01 (DCs and ESP+DCs *vs.* PBS control group); *p < 0.05 (DCs and ESP+DCs *vs.* ESP).

### Cytokine production

**Figure 4:**
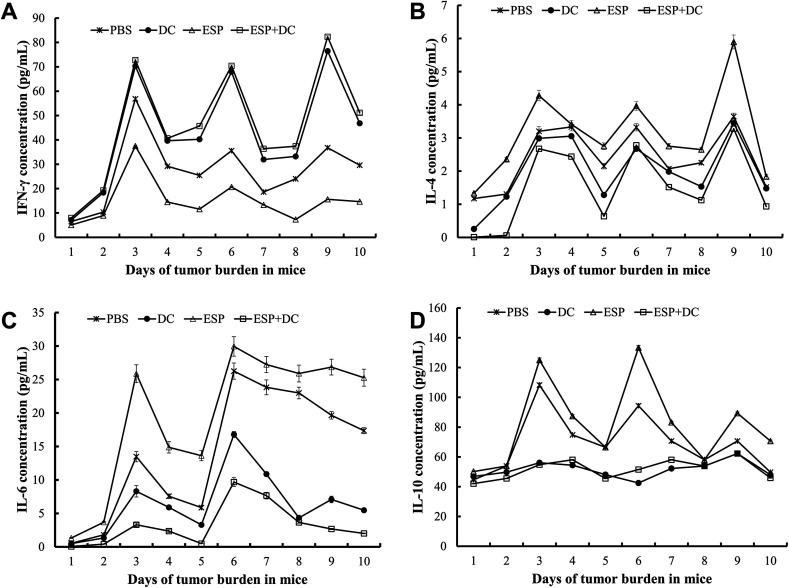
Cytokine content in serum of H22 hepatoma bearing mice treated with PBS, dendritic cells (DCs), excretory/secretory products (ESP) and DCs stimulated with ESPs. (A–D) IFN-γ, IL-4, IL-6 and IL-10 content in serum of H22 hepatoma bearing mice treated with PBS, DCs, ESP and DCs stimulated by ESPs during days 0–27.

H22 hepatoma bearing mice treated with DCs and DCs stimulated by ESPs showed a decrease in IL-4, IL-6, or IL-10 compared with their levels in the PBS and ESP groups (*p* < 0.05). The treatment with DCs and DCs stimulated by ESPs resulted in significantly increased expression of IFN-γ in comparison with H22 hepatoma bearing mice treated with PBS and ESPs only (*p* < 0.05). However, the reduction in IL-4, IL-6, IL-10, and the increase in IFN-γ were not significantly different in the mice treated with DCs and DCs stimulated by ESP (Fig. 4).

## Discussion

The antitumor effect of *T. spiralis* has been widely reported [16, 27, 45]. As a zoonotic nematode and foodborne parasite, *T. spiralis* infection leads to the suppression of the host immune response [13]. According to a previous study, *T. spiralis* antigens induce a mixed Th1/Th2 cytokine profile with a predominance of Th2 cytokines (IL-4, IL-6, and IL-10) increases [6, 35]. In this study, ML, ADs, and NBL ESPs were used as *T. spiralis* antigens. The results obtained in this study indicated that *T. spiralis* antigens from all three life stages of the parasite contributed to the development and maintenance of Th2 response. However, *T. spiralis* ESP had the ability to inhibit tumor growth. ESPs of *T. spiralis* contain some functional proteins such as proteinases, proteinase inhibitors, heat shock proteins, glycosidases, protein kinases, endonucleases, thymidylate synthase, migration inhibitory factors, nucleotide-metabolizing enzymes, prosaposin and GM2 activator protein, enolase, superoxide dismutase, caveolin, and prolactin, with a potential role in the invasion of the enterocytes, and establishment or maintenance of the nurse cell system [48]. During nurse cell formation, muscle cell differentiation, proliferation, cell cycle, and apoptosis are regulated by mitochondria-mediated factors from *T. spiralis* [2]. *Trichinella spiralis* infection causes a variety of changes in skeletal muscle cells and arrests the cell cycle of infected cells in G2/M for a long time [14]. A study showed that p53, a protein in ESPs, contributes to stagnation in the G1/G0 phase of the cell cycle [47]. Our hypothesis is that the antitumor effect of *T. spiralis* ESPs is not only due to the regulation of host immune response to Th2. If the immune response regulation has an effect on the antitumor immunity of the host, it impedes the antitumor application of *T. spiralis* ESPs. DCs are the most potent antigen-presenting cells in the host [15, 26] and mature cells can enhance the antitumor immune response [26]. CD11c is a specific antigen on the surface of mature DCs. During DCs maturation, the expressions of CD86 and MHC II increase, allowing DCs to activate the immune system [4]. Specific surface markers including CD11c^+^, CD86, and the MHCII family MHCII (I-A) were highly expressed in this work, indicating that mouse bone marrow-derived monocytes differentiated into mature DCs.

Mature DCs can strongly promote anticancer immunity and trigger Th1/Th17 cytokine secretion [8, 32, 33, 42, 46]. The close relationship between IL-4 and tumor progression has been investigated in several diseases [41, 42]. IL-6 overexpression is present in numerous cancer types, leading to the dysregulation of a plethora of cellular activities that generally promote tumor progression [37]. IL-10 is a classic anti-inflammatory cytokine that inhibits multiple DC functions, including maturation and production of proinflammatory cytokines [38]. In a cancer environment, IFN-γ promotes anti-tumoral immunity through its direct activity on both tumor and immune cells [11, 29, 46]. Our results showed that the production of IL-4, IL-6, and IL-10 in H22 hepatoma bearing mice treated with DCs stimulated by ESPs was significantly reduced, while the expression of IFN-γ was markedly increased, which was the opposite to the cytokine expression in the ESP group. However, no significant difference was observed in tumor inhibition rates and cytokine expression levels between the DCs group and the ESP+DCs group. Thus, our study suggested that ESPs had no immunological side effect to mature DCs in reducing tumor growth in the H22 hepatoma bearing mice.

Luo et al. suggested that *T. spiralis* ML ESPs induce apoptosis in H446 cells through a mitochondrial pathway, which may represent the antitumor mechanism [24]. ML ESPs, as parasitic products that are released during the chronic phase of infection, reduce the survival and slightly, but significantly increase the apoptosis of melanoma cells *in vitro* [43]. Our previous work also demonstrated that *T. spiralis* extracts induce apoptosis of K562 and H7402 cells, blocking their cell cycle in the G1 or S phase [44, 45].

Overall, our work demonstrated that the anti-tumor effect of *T. spiralis* did not affect the modulation of the immune response of mature DCs. Due to the complex immune response between the host and the parasite, the immunological safety of *T. spiralis* infection inducing antitumor effects needs further evaluation. Therefore, further studies that examine immunological changes are needed. Necrosis, apoptosis, and cell cycle change induced by ESPs of *T. spiralis* should not be neglected.
